# Artificial selection for male winners in the Siamese fighting fish *Betta splendens* correlates with high female aggression

**DOI:** 10.1186/s12983-019-0333-x

**Published:** 2019-08-08

**Authors:** A. Ramos, D. Gonçalves

**Affiliations:** Institute of Science and Environment, University of Saint Joseph, Rua de Londres 16, Macao, China

**Keywords:** Aggression, Artificial selection, Domestication, Mirror test, Sexual conflict

## Abstract

In Southeast Asia, males of the Siamese fighting fish *Betta splendens* have been selected across centuries for paired-staged fights. During the selection process, matched for size males fight in a small tank until the contest is resolved. Breeders discard losing batches and reproduce winner batches with the aim of increasing fight performance. We assessed the results of this long-term selection process by comparing under standard laboratory conditions male and female aggressive behaviour of one strain selected for staged fights (“fighters”) and one strain of wild-types. The aggressive response of adult fish was tested against their mirror image or a size-matched conspecific. Fighter males were more aggressive than wild-type males for all measured behaviours. Differences were not only quantitative but the pattern of fight display was also divergent. Fighter males had an overall higher swimming activity, performing frequent fast strikes in the direction of the intruder and displaying from a distance. Wild-type males were less active and exhibited aggressive displays mostly in close proximity to the stimuli. Females of the fighter strain, which are not used for fights, were also more aggressive than wild-type females. Aggressive behaviours were correlated across male and female fighter siblings, suggesting common genetic and physiological mechanisms to male and female aggression in this species. The study further shows that results were largely independent of the stimulus type, with the mirror test inducing similar and less variable responses than the live conspecific presentation. These results suggest that selection for male winners co-selected for high-frequency and metabolic demanding aggressive display in males and also enhanced female aggression, opening a wide range of testable hypothesis about the ultimate and proximate mechanisms of male and female aggression in *B. splendens*.

**Electronic supplementary material:**

The online version of this article (10.1186/s12983-019-0333-x) contains supplementary material, which is available to authorized users.

## Background

Artificial selection and experimental evolution are powerful tools for testing proximate and ultimate causes of behaviour. These manipulations may be carried out under controlled conditions in laboratory or natural settings or arise from unintended natural experiments. Domestication is a particularly interesting example as it usually combines adaptation to captive conditions and selective breeding for particular traits [1]. The comparison between domesticated and wild-type strains was key to Darwin’s theory of natural selection [2, 3] and since then it has enriched our understanding of the general mechanisms of behaviour and its evolutionary principles [4, 5]. Studying organisms that have been under strong artificial selection for particular behavioural traits may facilitate the identification of genetic and physiological mechanisms underlying the expression of that behaviour (e.g. [6]) and, because selection for one particular behaviour usually co-selects for other traits, either behavioural or morphological, uncover gene epistatic and pleiotropic effects [7].

Domestication is usually associated with selective breeding for tame behaviour but in a few species animals have been selected for high aggression either for cultural (e.g. fighting bulls, [8] fighting dogs, [9]) or research (e.g. fruit fly, [10]; mice, [11]) purposes. In most of these models, males are the target of selection because of their inherently higher levels of aggression as compared to females. However, because males and females share the vast majority of the genome [12], the directional selection for aggression in males may lead to increased aggression in females if female and male aggression are genetically correlated. The study of these systems may thus provide relevant information not only on the underlying genetic and physiological mechanisms of aggression but also on how sexual conflict may shape the evolution of aggressive behaviour [13, 14].

The Siamese fighting fish *Betta splendens* is a particularly interesting model to investigate proximate and ultimate behavioural questions. This species has been selected for body colour, fin patterns and behaviour, and the wide variety of currently available phenotypes makes it one of the most important species in the freshwater ornamental fish industry. Its aggressive behaviour has been well-characterized [15] and female-female aggression occurs naturally [16]. Complex social modulation of aggressive displays has been documented, with both audience [17–19] and eavesdropping [20, 21] effects described. Of relevance, wild-type populations, presumably similar to the original populations that originated the domesticated varieties, can still be found in remote areas of Southeast Asia, allowing for comparative studies between the wild-type and domesticated strains.

Fighting strains of *B. splendens* have been under selection for more than six centuries [22, 23], originating short-fin varieties known in Thailand as “Plakat Morh”. During the selection process, matched for size males are paired in small tanks for staged fights. Fights can last several hours and after resolution fighters are discarded and the siblings of male winners are selected for breeding. Breeders try to improve the fighting lines by cross-breeding varieties that have specific morphologic and behavioural traits but the selection process is centred in the fight outcome and not on behavioural displays.

In a previous study, the male aggressive behaviour of domesticated and wild-type *B. splendens* was compared using a combination of mirror and video playbacks tests and live conspecific presentations [24]. The authors showed that results were context dependent, with differences in aggression between wild-type and short-finned domesticated varieties (results from a fighter and fancy short-finned variety where pooled together to increase sample size) detected during live conspecific trials but not when using mirror/video playback tests. Further, the same fighter and wild-type strains showed differences in male cortisol response to unfamiliar environments, with wild-types, but not fighters increasing cortisol levels [25]. Taken together, the results from these studies suggest that selection for winning has modified aggressive behaviour and physiological responses in *B. splendens*.

However, the possible impact of the selection process on females, who are not used in fights, remains to be investigated. Selection targeting males may have induced changes in female behavior and physiology via shared genetic mechanisms and possibly drive sexual conflict. A detailed characterization of the behavioural consequences of directional selection for male winners in both sexes is thus relevant to understand proximate and ultimate mechanisms of aggression in *B. splendens*.

In this study, we compared the aggressive behaviour of a fighter and a wild-type strain to investigate: (1) the impact of selection for winners in male aggressive displays; (2) the possibility of correlated responses in the aggressive behaviour of females, which are not directly targeted by the selection process; (3) the possible variation in the aggressive response of males and females of both strains to two different types of aggression-eliciting stimulus, a mirror image and a size-matched conspecific.

## Methods

### Study species

**Fig. 1:**
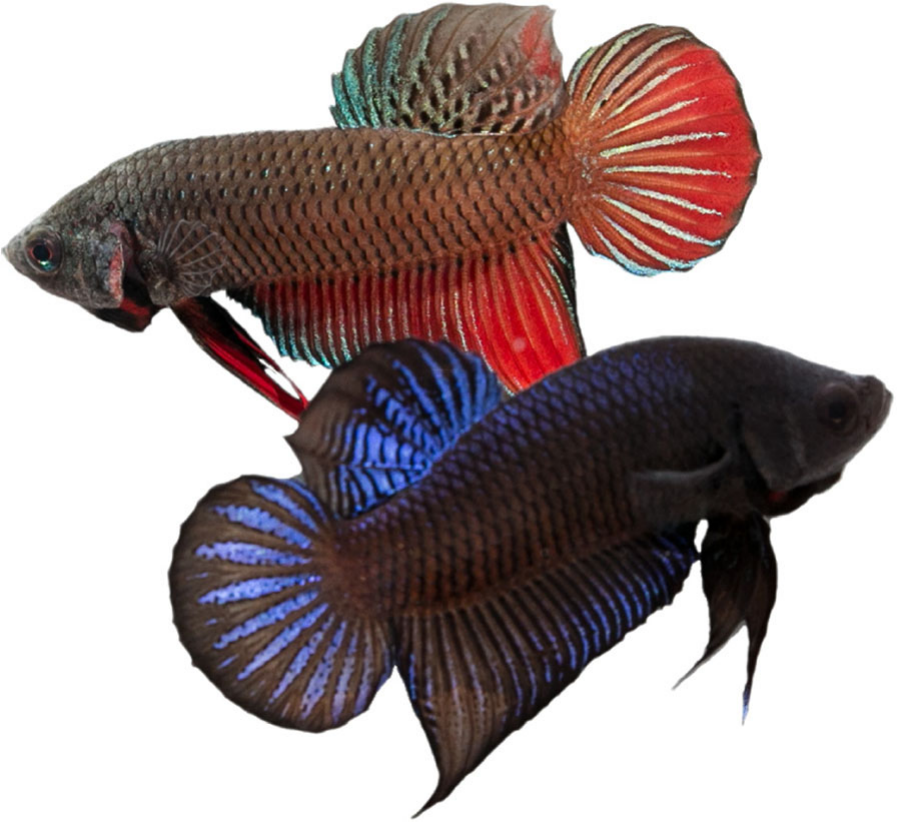
A wild-type (top) and fighter (bottom) male *B. splendens* of the strains used in the experiments

We compared the behaviour of a wild-type and a fighter strain of *B. splendens* (Fig. 1). Species identity was confirmed for all F0 fish by sequencing the ITL1 and COI genes following [26, 27] (data not shown). Wild-types had been captured in Chiang Rai Province, Thailand, and were acquired from a local and authorized retailer. An initial mix-sex group of approximately 60 individuals was used to start a founding population in the lab. Ten pairs of F1 males and females from different families (different F0 parents) were mated to originate the F2 fish, with an approximately similar number of fish per family used in the experiments. Fighter fish were acquired from a reliable and authorized commercial retailer from Bangkok, Thailand. To start the fighter line, 12 sibling males from one breeder and 12 sibling females from another breeder were used (F0 fish), replicating a mating scheme commonly used by local breeders where sibling males of a winner are mated with sibling females from another winner. From the resulting F1, and similarly to the mating scheme for wild-types, males and females from different families were crossed to originate the F2 fish used in this study. Under laboratory conditions, the artificially selected traits associated with winning were probably under relaxed selection because there was no attempt to further select winners. The potential decrease in expression of the winning-related traits was minimized by limiting the initial genetic pool to two fighter families and by testing animals after only two generations. For breeding, a tank (50 W X 30D X 25H cm) without substrate or aeration, with shelters and aquatic plants for the female to hide, and with the water conditioned by adding one large indian almond leaf was used. The male was added to the tank and the female to a separate transparent box (10 X 10 X 10 cm) inside the larger tank. They were allowed to have only visual contact until the male built a bubble nest. The female was then carefully removed from the box with a hand net and released and spawning usually occurred within 24 h. After spawning, the female was removed from the tank and the male was allowed to provide parental care to the eggs and larvae for a period of 5 days. The male was then removed and if necessary the brood was divided into more similar tanks to achieve an approximate raising density of 1 fish per 3 L. An external filter was added when fish were approximately 2 month old. We used 8 ± 1 months old males and females of both strains for all experiments. Standard length (cm) of fish used in the experiments were as follows (x̄ ± S.D., fighter males = 3.78 ± 0.02; fighter females = 3.54 ± 0.19; wild-type males = 3.42 ± 0.05; wild-type females = 3.37 ± 0.18). At this stage, sex-ratio in the stock tanks was approximately 1:1. Conditions in the stock and experimental tanks were similar, with temperature being kept at 28 ± 1 °C, the photoperiod set to 12:12 L:D and tank water supplied by a reverse osmosis system. Animals were fed once a day with a mixture of dry (tubifex worms and pellets from different brands) and live (adult *Artemia*) food.

### Behavioural recordings

Prior to being tested, fish were isolated in individual tanks (28 W X 14D X 20H cm) for 1 week without any visual contact with other fish. Tank walls were lined on the outside with styrofoam plates, except the wall through which observations were conducted. Animals were randomly assigned to one of three treatments: 1) mirror; 2) live conspecific and; 3) glass (control) test. There were no significant size differences in standard length between treatments for fish of the same strain and sex (one-way ANOVA: fighter males, *F*_*2,46*_ = 0.85, *P* = 0.432; fighter females, *F*_*2,46*_ = 1.46, *P* = 0.244; wild-type males, *F*_*2,46*_ = 0.79, *P* = 0.460; wild-type females, *F*_*2,46*_ = 0.42, *P* = 0.660). For the mirror and control trials, a mirror or a same size transparent glass, respectively, were placed adjacent to one of the shorter walls of the test tank. For the live conspecific test, an individual tank containing a matched for size (standard length coefficient of variation < 5%) conspecific of the same sex and strain was positioned to the top end of the tank of the focal animal. An opaque partition between the two tanks that did not allow the animals to see each other was removed to initiate the trial. The side of presentation (left or right) was randomized for all treatments. Fish used as models in the live conspecific test were also isolated for 1 week prior to being used and were not tested as focal animals. A subset of animals was also tested in a second control, an empty tank placed adjacent to one of the top ends of the test tank. Because no differences in behaviour of focal animals were recorded between this and the glass control (data not shown), it was decided to include only the latter in the analysis.

Trials had a duration of 15 min and were video recorded at 60 cm from the test tank through an opening in an opaque partition using a Sony HDR-PJ670 Handycam. Mirror and conspecific trials began after the first aggressive display was recorded (usually within the first minute) and the control trials began immediately after the glass was placed. Behavioural displays (Table 1) were quantified from video using JWatcher V1.0 (Macquarie University and UCLA). For frontal and lateral displays, total duration was highly correlated with the frequency of the behaviour (Spearman correlation coefficient, frontal display, *r*_*s*_ > 0.89, *N* = 184, *P* < 0.001; lateral display, *r*_*s*_ > 0.93, *N* = 184, *P* < 0.001) and it was decided to use only the frequency of the behaviours for the analyses. Total travelled distance and time spent within one body distance to the stimuli was calculated using UMA Tracker (http://ymnk13.github.io/UMATracker/).

**Table 1:** Description of the aggressive behaviours quantified. An aggression score was calculated as the average frequency of these displays

	Behaviour	Description
	Frontal display	Faces the opponent with unpaired fins and/or opercula extended. Body in an angle between 45 to 90 degrees to the opponent.
	Lateral display	Exposes the flank to the opponent with unpaired fins and/or opercula distended. Body in an angle of less than 45 degrees to the opponent.
	Charge	Swims fast in the direction of the opponent.
	Caudal swing (tail beats)	Waves the caudal fin towards the opponent side.

To test the hypothesis that male and female aggressive behaviour could be correlated, one male and female sibling pair from each of 10 different families (different father and mother) of the fighter strain raised in separate aquaria were tested with the mirror presentation, following the above procedures. Males were 3.87 ± 0.12 cm and females 3.65 ± 0.14 cm in standard length. An aggression score was calculated as above and the correlation between male and female aggressive behaviour computed. It was not possible to run the same analysis for the wild-type strain because a sufficient number of sibling pairs raised in separate tanks were not available.

### Statistical analyses

Aggression-related variables (Table 1) were all significantly correlated between each other (*r*_*s*_ > 0.33, *N* = 111, *P* < 0.001). To minimise the number of comparisons between groups, an aggression score was first calculated as the average frequency of frontal and lateral displays, caudal swings and charges. Behavioural differences between strains (wild-type and fighter), sex (male, female), treatment (control, mirror, glass) and the possible interaction between these factors were tested with a three-way general linear model (glm). Only two levels of the factor treatment (mirror and conspecific) were used for analysis of the aggression score as aggressive behaviors were not displayed in the control (glass) treatment. Specific comparisons within or between levels of the factors were tested with planned contrasts. The aggression score was squared root-transformed while the frequency of air breathing and the total distance travelled were log-transformed to comply with heteroscedasticity assumption. Effect sizes on glm analysis are reported by partial ETA squared (η_p_^2^).

To test for possible differences in the variability of the aggressive response to the mirror and conspecific treatment, a Levene’s test on non-transformed values of the aggression score was applied separately to each of the four experimental groups (i.e. fighter males, fighter females, wild-type males and wild-type females). These statistical analyses were performed with IBM SPSS Statistics version 25.

To test whether the overall behaviour of a group differed from the other groups the quadratic assignment procedure (QAP) correlation test was applied. In this procedure, a Pearson correlation matrix for the different behaviors is calculated for each group and compared with the matrix of the other groups. The QAP null-hypothesis indicates that there is no association between matrices and thus correlation matrices are considered different when the *P*-value is higher, not lower, than 0.05 ([27], for an example see [28]). QAP analysis were performed with the Ucinet for Windows software [29]. The Benjamini-Hochberg procedure [30] was applied to correct for the multiple Pearson correlation tests within groups.

## Results

**Fig. 2:**
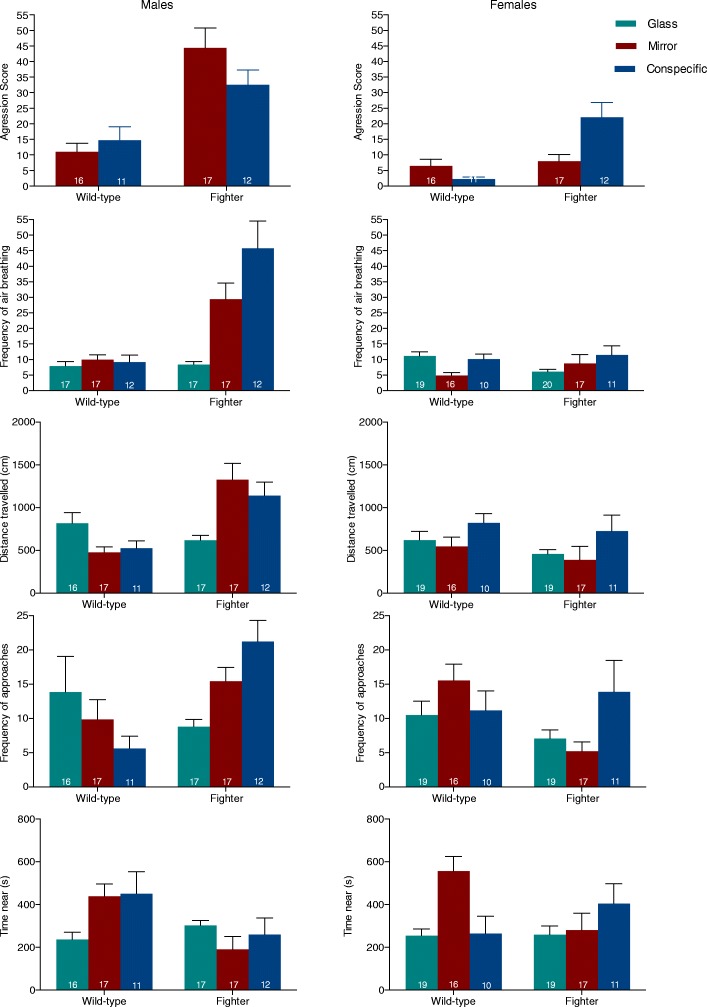
Aggression score and activity behaviours quantified for wild-type and fighter males (left) and females (right). For the aggression score only the mirror and conspecific treatments are included as fish did not display these behaviours during glass (control) trials. Numbers in columns indicate *N*. Means ± S.E. are shown

An overall analysis of the results shows that fighters had an aggression score three times higher than wild-types (Table 2, Fig. 2). Differences in aggression between males and females were of a similar magnitude, and higher in males.

**Table 2:** Three-way ANOVA results with factors sex (male and female), strain (fighter and wild-type) and treatment (conspecific, mirror and control) and interaction between the factors. For the aggression score, only two levels of the factor treatment (conspecific and mirror were included as aggressive behaviours were absent during control trials. Values for the F-statistic and partial eta-squared (η_p_^2^) are reported. * *P* < 0.05, ** *P* < 0.01, ***P < 0.001

Variable		Strain	Sex	Treatment	Strain*Sex	Strain*Treatment	Sex*Treatment	Sex*Strain*Treatment
Aggressive displays								
Aggression score	F η_p_^2^	*F*_*1,104*_ = 43.61*** 0.30	*F*_*1,104*_ = 32.03*** 0.24	*F*_*1,104*_ = 0.27 < 0.01	*F*_*1,104*_ = 3.22 0.03	*F*_*1,104*_ = 1.29 0.01	*F*_*1,104*_ = 1.39 0.01	*F*_*1,104*_ = 7.76** 0.07
Activity								
# Air breathing	F η_p_^2^	*F*_*1,174*_ = 13.10*** 0.07	*F*_*1,174*_ = 15.17*** 0.08	*F*_*2,174*_ = 4.80** 0.05	*F*_*1,174*_ = 21.80*** 0.11	*F*_*2,174*_ = 6.49** 0.07	*F*_*2,174*_ = 6.96** 0.07	*F*_*2,174*_ = 1.10 0.01
Time near	F η_p_^2^	*F*_*1,171*_ = 4.44* 0.03	*F*_*1,171*_ = 0.42 < 0.01	*F*_*2,171*_ = 3.55* 0.04	*F*_*1,171*_ = 1.84 0.01	*F*_*2,171*_ = 6.06** 0.07	*F*_*2,171*_ = 1.34 0.02	*F*_*2,171*_ = 2.42 0.03
# Approach	F η_p_^2^	*F*_*1,174*_ = 0.29 < 0.01	*F*_*1,174*_ = 1.44 0.01	*F*_*2,174*_ = 1.10 0.01	*F*_*1,174*_ = 8.22** 0.05	*F*_*2,174*_ = 6.11** 0.07	*F*_*2,174*_ = 0.09 < 0.01	*F*_*2,174*_ = 3.41* 0.04
Distance travelled	F η_p_^2^	*F*_*1,174*_ = 0.23 < 0.01	*F*_*1,174*_ = 5.38* 0.03	*F*_*2,174*_ = 0.67 0.01	*F*_*1,174*_ = 19.67*** 0.10	*F*_*2,174*_ = 0.82 0.01	*F*_*2,174*_ = 3.22* 0.04	*F*_*2,174*_ = 1.45 0.02

These differences were relatively independent of stimulus type (mirror or conspecific) and, accordingly, total variance, as given by η_p_^2^, was mostly explained by strain and sex rather than treatment (Table 2).

### Males

**Fig. 3:**
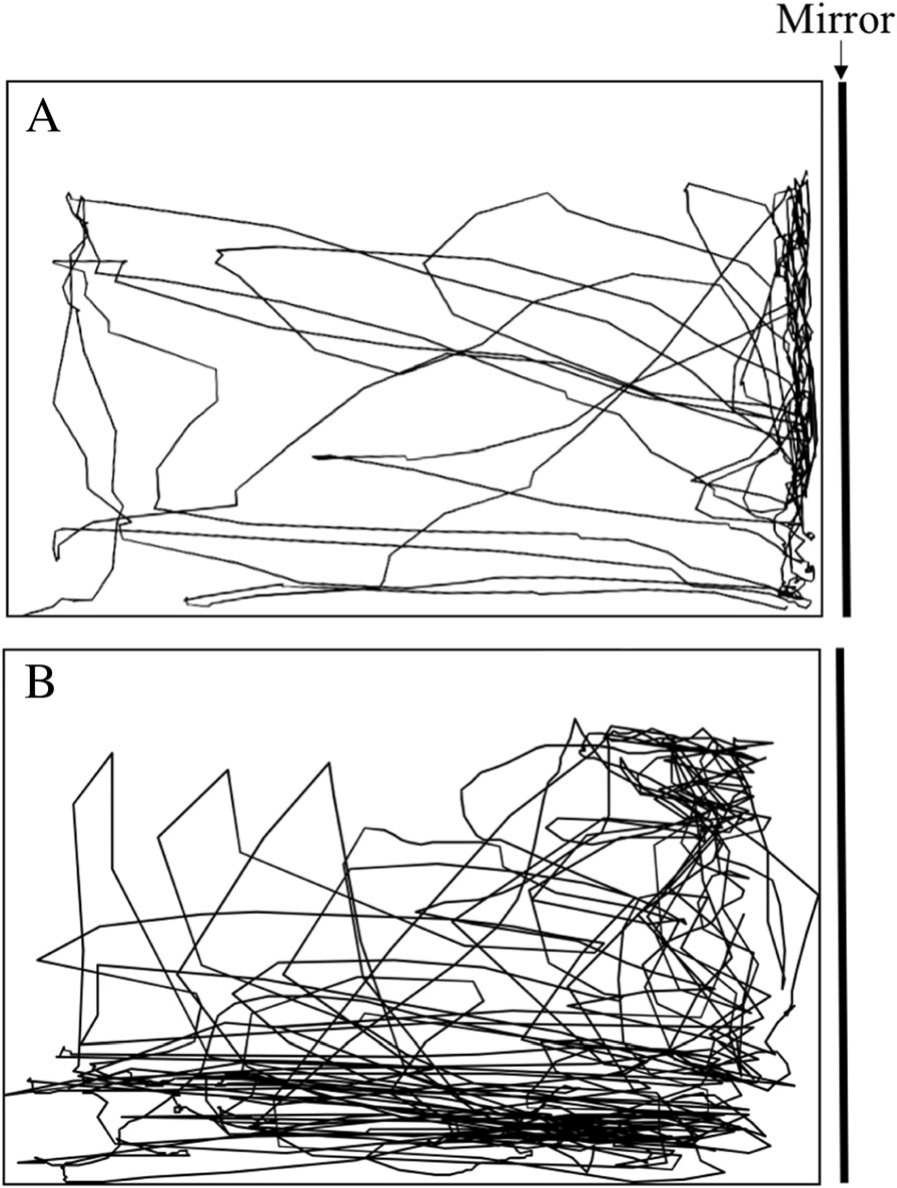
Representative 2D side-view tracks of a wild-type (**a**) and fighter (**b**) male during mirror trials. Tracks from the fish with a total distance travelled closest to the strain average were used

**Fig. 4:**
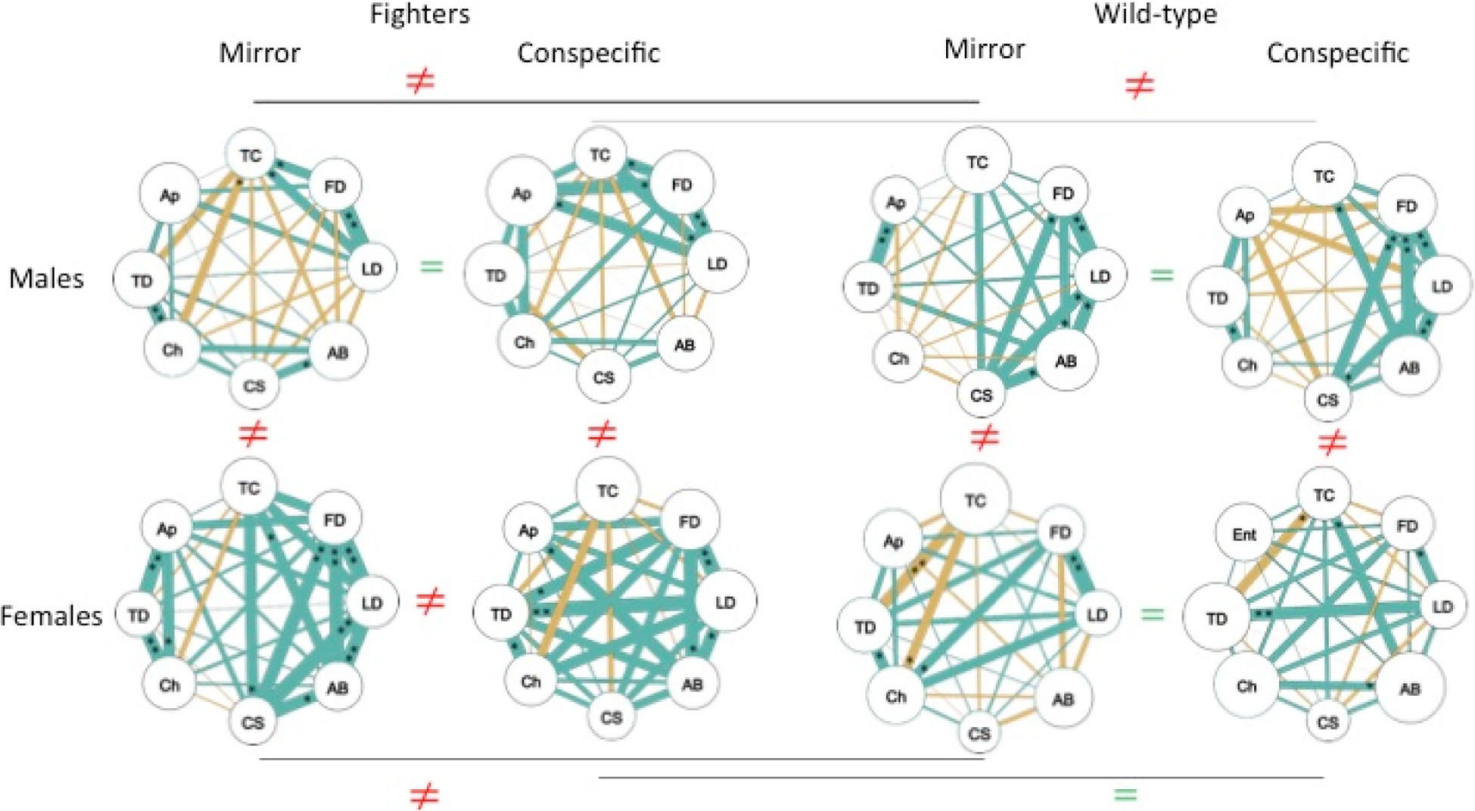
Behavioural correlation networks for each of the aggression-eliciting groups. The diameter of circles represents the frequency of the behaviour, total distance travelled or time spent close to stimuli (normalized between 0 and 1). FD – frequency of frontal display; LD – frequency of lateral display; Ch – frequency of charge; CS – frequency of caudal swing; Ap – frequency of approach; AB – frequency of air breathing; TD – total distance travelled; TC – time spent close to stimuli. Distinct (≠) and similar (=) correlation network patterns among groups are indicated. Lines within groups represent Pearson’s correlation coefficients (*r*), with line thicknesses proportional to *r* value and positive / negative correlations indicated by line colour (green / yellow, respectively). Asterisks indicate significant correlations after adjustment for multiple comparisons: *P < 0.05; **P < 0.01 and ***P < 0.001

Planned contrasts comparing only males confirmed that the aggression score was higher for fighter than wild-type males (*P* < 0.001, η_p_^2^ = 0.26; Fig. 2, see Additional file 1: Figure S1 for individual aggression measures). The frequency of air breathing, a correlate of metabolic activity [31], was over three times higher in fighter than in wild-type males during mirror (*P* = 0.001, η_p_^2^ = 0.07) or conspecific (*P* < 0.001, η_p_^2^ = 0.14) trials but not during control trials (*P* = 0.252, η_p_^2^ = 0.01). Further, while fighters increased air breathing frequency during conspecific (*P* < 0.001) or mirror (*P* = 0.001) trials, as compared to controls (η_p_^2^ = 0.13), wild-types did not (conspecific, *P* = 0.918; mirror, *P* = 0.234); η_p_^2^ = 0.01). During the conspecific and mirror trials, the frequency of air breathing was positively correlated with the aggression score and activity-related variables (Spearman correlation coefficient, *r*_*s*_ > 0.37, *N* = 58, *P* < 0.004), except with the time spent close to the stimuli (*r*_*s*_ = − 0.16, *N* = 57, *P* = 0.235). Fighter males spent less time in close contact with the opponent (conspecific, *P* = 0.055, η_p_^2^ = 0.02; mirror, *P* = 0.003, η_p_^2^ = 0.05), approached more often the area close to the stimuli (conspecific, *P* < 0.001, η_p_^2^ = 0.07; mirror, *P* = 0.120, η_p_^2^ = 0.01) and travelled longer distances (conspecific, *P* = 0.005, η_p_^2^ = 0.05; mirror, *P* = 0.014, η_p_^2^ = 0.03) than wild-types (Fig. 2). Again, these differences were only evident during aggression-elicited trials but not during controls (*P* > 0.095). This was explained by the different approach of males of the two strains to the mirror/conspecific challenge. Typically, fighter males would swim back and forth, approaching or charging the stimuli very often but avoiding prolonged contact with the opponent and displaying from a distance of a few cm (Fig. 3). Wild-type males, on the other hand, spent most of the trial time displaying within short distance. In fact, while wild-type males increased the time spent in close contact with the stimuli during the mirror (*P* = 0.015) and conspecific (*P* = 0.022) trials, as compared to controls (η_p_^2^ = 0.04), fighter males did not (*P* > 0.169, η_p_^2^ = 0.01; Fig. 2). This different pattern of fighting and activity during aggression-eliciting trials for males of the two strains was confirmed by QAP correlation analysis that revealed a different behavioural network correlation pattern for the mirror (*r* = 0.09, *P* = 0.276) and conspecific (*r* = 0.14, *P* = 0.222) treatment (Fig. 4).

### Females

Although females have not been directly selected for fighting, differences in aggressive behaviour between strains were similar to those observed for males. Fighter females were more aggressive than wild-type females (*P* = 0.001, η_p_^2^ = 0.10; Fig. 2, see Additional file 1: Figure S1 for individual aggression measures). On the contrary, activity-related variables did not differ between females of the two strains (frequency of air breathing, *P* = 0.462, η_p_^2^ < 0.01; time spent close to opponent, *P* = 0.596, η_p_^2^ < 0.01; frequency of approaches to the area close to the stimuli, *P* = 0.102, η_p_^2^ = 0.02), with the exception of the total distance travelled (*P* = 0.006, η_p_^2^ = 0.04), higher in wild-type females (Fig. 2).

Metabolic activity, as inferred by the frequency of air breathing, was positively correlated with the aggression score and all activity-related variables (*r*_*s*_ > 0.29, *N* = 54, *P* < 0.036).

QAP correlation analysis suggested that differences in aggressive behaviour between females of the two strains were mostly quantitative rather than qualitative, as supported by an overall behavioural network correlation pattern similar in the conspecific treatment (*r* = 0.42, *P* = 0.010) and marginally different for the mirror treatment (*r* = 0.31, *P* = 0.070; Fig. 4).

### Males vs females

Although females displayed frequent aggressive behaviours towards the conspecific or mirror image, males were clearly more aggressive than females, both in the fighter (*P* < 0.001, η_p_^2^ = 0.21) and wild-type (*P* = 0.008, η_p_^2^ = 0.07) strain (Fig. 2, see Additional file 1: Figure S1 for individual aggression measures).

Fighter males were also more active, approaching the stimuli more often (*P* = 0.004, η_p_^2^ = 0.05) and travelling longer distances (*P* < 0.001, η_p_^2^ = 0.12), probably contributing to the higher frequency of air breathing (*P* < 0.001, η_p_^2^ = 0.18) in males than in females of this strain. Again, these differences were only evident during aggression-eliciting trials and not during controls (*P* > 0.071). On the contrary, activity-related variables were generally similar for male and female wild-type (*P* > 0.141).

Differences between males and females in behaviour were confirmed by the QAP behavioural network correlation analyses which differed for the two strains and for the two aggression-eliciting stimuli (fighter mirror, *r* = 0.29, *P* = 0.084; fighter conspecific, *r* = 0.14, *P* = 0.307; wild-type mirror, *r* = 0.10, *P* = 0.315; wild-type conspecific, *r* = 0.06, *P* = 0.327; Fig. 4).

**Fig. 5:**
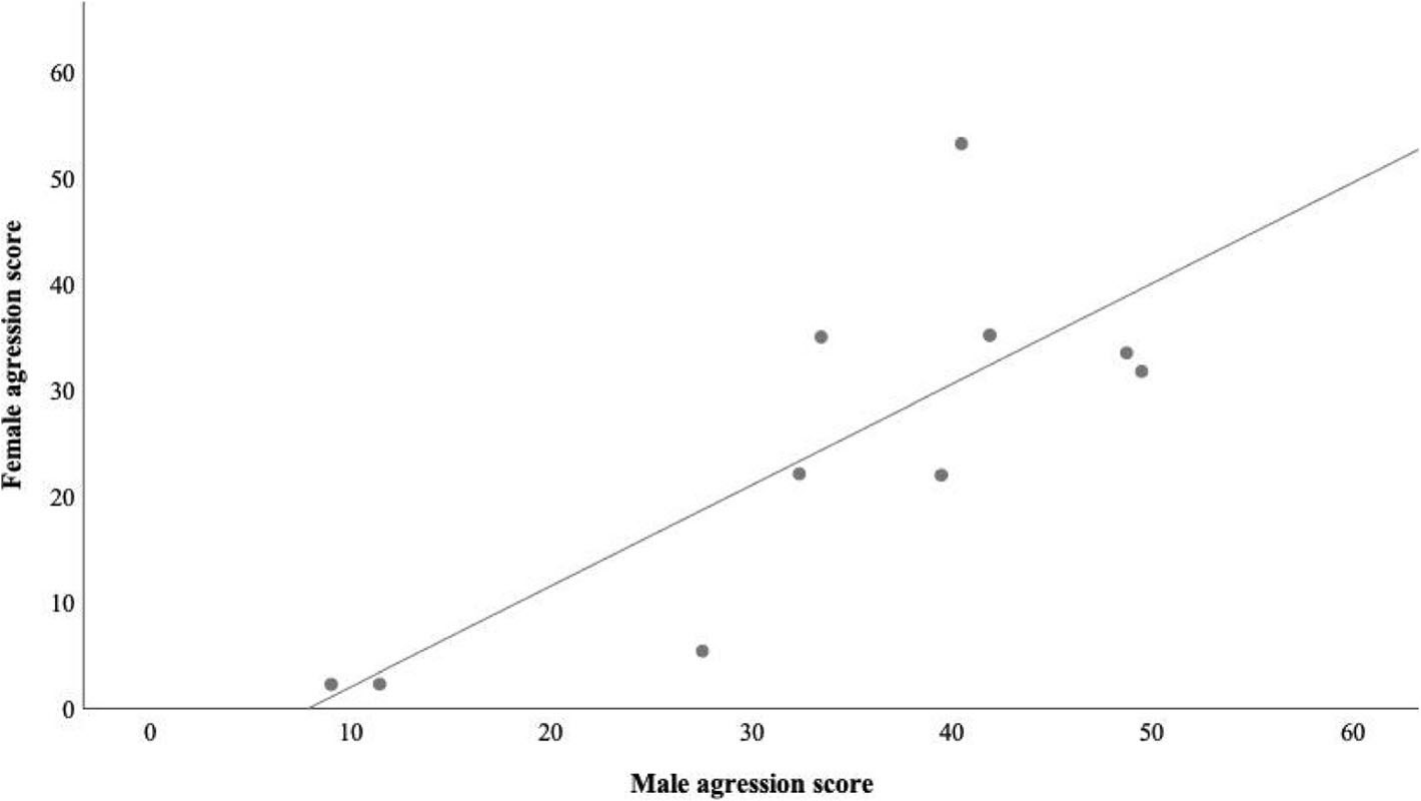
Aggression scores for 10 pairs of male and female fighter siblings from different families. Spearman correlation coefficient: *r*_*s*_ = 0.73, *N* = 10, *P* = 0.021

To further assess for a possible correlation between male and female aggression, 10 pairs of male and female fighter siblings from different families, raised in separate aquaria, were tested against their mirror image. The frequency of male and female aggressive behaviours were all positively and significantly correlated (frontal displays, *r*_*s*_ = 0.82, *P* = 0.007; lateral displays, *r*_*s*_ = 0.65, *P* = 0.049; caudal swings, *r*_*s*_ = 0.89, *P* = 0.001), with the exception of the frequency of charges (*r*_*s*_ = 0.55, *P* = 0.102), resulting in an overall significant correlation between male and female aggression scores (*r*_*s*_ = 0.73, *P* = 0.021; Fig. 5).

### Conspecific vs mirror

Behavioural differences found between males of the two strains were independent of the stimulus type. In fact, planned contrasts confirmed that the response of fighter (*P* > 0.120) and wild-type (*P* > 0.168) males to the mirror and conspecific stimulus was comparable for the aggression score and all activity-related behaviours. This generally similar pattern of response to the mirror and conspecific challenge resulted in comparable behavioural network correlation patterns between treatments both for fighter (*r* = 0.77, *P* < 0.001) and wild-type (*r* = 0.66, *P* = 0.002) males (Fig. 4).

For females, a different pattern was recorded for fighter and wild-type strains. Wild-type females presented a similar response to the two stimuli (*P* > 0.078), except for the time near the stimulus, higher in the mirror treatment (*P* = 0.006, η_p_^2^ = 0.07). On the other hand fighter females were more aggressive (*P* = 0.006, η_p_^2^ = 0.07) and active (approaches to the stimulus area, *P* = 0.033, η_p_^2^ = 0.03; total distance travelled, *P* = 0.027, η_p_^2^ = 0.03) towards the conspecific than towards their mirror image. These results were aligned with the QAP tests for the behavioural correlation networks as fighter females differed between the mirror and conspecific treatment (*r* = 0.24, *P* = 0.136), while wild-type females did not (*r* = 0.46, *P* = 0.010) (Fig. 4).

In the conspecific test, the aggressive response of the focal fish may be expected to be more variable as compared with the mirror test because it depends on the dynamics of the interaction with another fish. This was only partially confirmed as the aggression score was more variable towards the conspecific than towards the mirror image only in fighter females (*P* = 0.015; *P* > 0.058 for the other groups).

## Discussion

Domestication is a type of experimental evolution usually associated with intentional selective breeding and where control over the selection process is low [1]. Although such design is less powerful than careful artificial selection experiments performed under controlled conditions, data gathered from the comparison between wild-type and domesticated strains can be most informative for the understanding of evolutionary processes, in particular because they often provide the only way to access long-term selection processes that cannot be replicated in laboratory settings. That is the case of selection for male winners in *B. splendens* for two reasons: first, breeders select the strains for reproduction from the outcome of paired-fights where fish can be injured or even die and, for ethical reasons, this selection process cannot be replicated for research purposes; second, selection for winners has been going on for centuries, limiting the possibility for a similar approach under controlled laboratory settings. The comparison of wild-type and fighter strains of this species thus allows a unique opportunity to test hypothesis on the proximate and ultimate mechanisms underlying aggression in fish.

The first prediction of our study was that selection for winning staged-fights would have enhanced aggressive displays in male *B. splendens*. This prediction was fully verified as differences were evident for all measured aggressive behaviours and for both aggression-eliciting contexts (mirror image and conspecific presentation). The results partially parallel a previous study in this species that had demonstrated enhanced aggression in a fighter strain as compared with a wild-type strain [24]. However, in that study differences were evident only during conspecific presentations (“mutual-viewing test”) and not when aggression was elicited by a combination of mirror images and video playbacks of conspecifics. Because the responses to mirror images and video playbacks, which need to be properly validated [32], were combined, a direct comparison with the results that we have obtained for the mirror test is not possible. Also, the same study compared differences between strains using aggression scores derived from a principal component analysis (PCA) and not individual behaviours, further limiting a direct comparison with our results. Still, the fact that both studies, which used different strains of wild-type and fighter animals, concluded for a higher aggressiveness of fighters, in line with our initial prediction, strongly suggest that differences between strains are the result of the selection process for winning and not of other uncontrolled factors. It should be highlighted that the selection criteria used by breeders is winning/losing and not the frequency/intensity of aggressive displays during the staged-paired fights. The fact that fighters exhibited a higher frequency of aggressive behaviours suggest that selection for winning co-selected for more frequent aggressive displays. One hypothesis is that males that display more frequently during fights will have a higher probability of winning, and indeed this has been previously demonstrated for *B. splendens* [33].

**Fig. 6:**
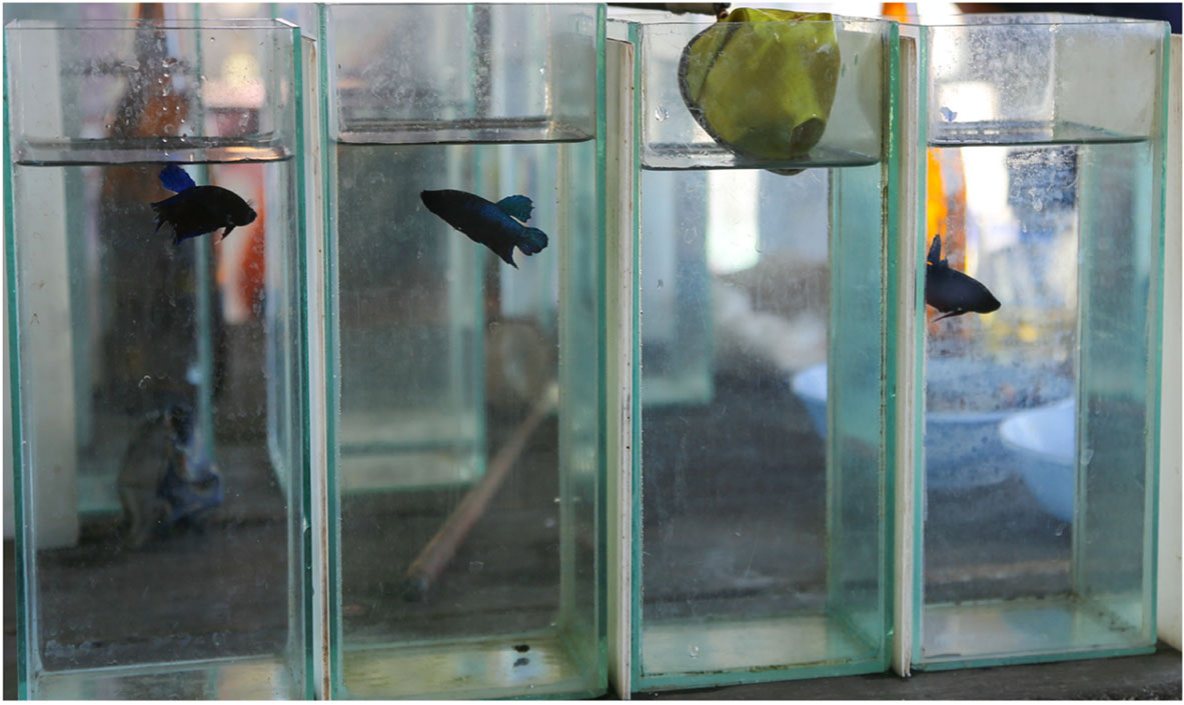
Example of tanks used for staged pair-fights in fighting rings across Southeast Asia

In addition to quantitative differences in the frequency of aggressive behaviours, the approach of fighter and wild-type males to the aggression challenge was also strikingly different. Typically, wild-type males would stay for most of the trial displaying in close contact with the mirror image or live conspecific tank. Fighters, on the other hand, would repeatedly swim back and forth, darting into the opponent’s direction and displaying from a distance. Accordingly, the pattern of the behavioural correlation network of wild-type and fighter males differed both for the mirror and conspecific treatment. The higher swimming activity and frequency of aggressive displays of fighter males was reflected in an increased frequency of surface air breathing, a correlate of oxygen consumption and metabolic activity [31]. These results may be understood in light of the selection process for winners. Staged fights take place in small, tall containers, without any hiding places or escape routes (Fig. 6). Observations from videos taken in fighting rings show that fish try to gain a position near the surface to block the opponent from breathing air (personal observations). Fights can last for several hours and fish that are able to inflict more damage to the opponent usually win the fight, with its siblings being selected for breeding. Thus, compared to wild-types, fighters seem to take a more cautious approach during fights, keeping a safe distance from the opponent and striking frequently with fast swims, probably because this strategy may be more efficient in the context of staged-fights as compared with the fights in the natural environment.

Second, we tested the hypothesis that female aggressive behaviour could have been affected by the selection process targeting males. Although female aggression is common in many species and plays a role in securing limited resources [34], it is still unclear whether female and male aggression share the same proximate mechanisms or if there are male and female-specific physiological modulators of aggression. Likewise, there is still no clear understanding of whether female aggression is primarily the result of a correlated evolutionary response to selection acting on male aggression or of direct selection acting on females [35]. Studies on this topic offer contradictory evidence. For instance, selection for high male or female aggression in fruit flies [36] and mice [37, 38] did not affect aggression levels in the other sex, thus suggesting that male and female aggression are not genetically correlated or that there were genetic compensation mechanisms [39]. On the contrary, exogenous androgen administration in fish [40] and birds [35] has been shown to enhance aggression both in males and females and comparative analyses demonstrate a covariation of testosterone in the two sexes [35, 41], suggesting shared mechanisms to male and female aggression.

Fighter females, which are not used in staged-fights, were more aggressive than wild-type females. Differences were observed for aggressive displays and not for activity-related measures and, unlike for males, seemed to be mostly quantitative rather than qualitative as the behavioural correlation networks of females of the two strains were similar for the conspecific test and marginally different for the mirror trials. The higher aggression of fighter females may be the result of common genetic and physiological mechanisms to male and female aggression but other alternative explanations associated with the domestication process can be considered. For example, the increase in female aggression in fighters may represent an adaptation to captivity with more aggressive females being more successful. These fish are raised under mixed high-density groups and it is possible that more aggressive females have higher survival rates, better access to food and an overall better body condition, being preferentially chosen by breeders to be paired with males for reproduction. Under this hypothesis, the high levels of female aggression in fighters could have resulted from an independent and unintended directional selection process, targeting either common or independent physiological mechanisms to those of male aggression. However, this hypothesis would not explain the observed positive correlation between male and female aggression in fighter siblings. In fact, male and female aggressive behaviour from sibling pairs of different fighter families raised separately were correlated, supporting the hypothesis that the process of selection for male aggression has resulted in a correlated response in females. This claim is further supported by anecdotical evidence gathered from *B. splendens* breeders in Thailand that report generally higher levels of aggression in females from more aggressive fighter strains (personal communication). As males and females share the vast majority of their genomes [12], it is reasonable to hypothesise that directional selection for high male aggression has produced similar physiological and phenotypic changes in females. In *B. splendens* it was shown that a fighter strain had a lower cortisol response to an unfamiliar context than a wild-type strain [25] and low cortisol levels have been associated with high-aggression in fish (e.g. [42]). It is thus possible that selection for male winners has reduced the activation of the stress-axis, explaining behavioural differences in male behaviour across strains and the correlated female fighter response. Taken together, our results suggest shared mechanisms to male and female aggression in *B. splendens* and a correlated genetic response in females to selection targeting males. However, further studies on the genetic and physiological mechanisms of male and female aggression and on the fitness consequences of high levels of female aggressiveness in different strains of *B. splendens* are needed.

The expression of female aggressive behaviour was significantly lower than in males not only in the fighter strain but also in wild-types. This suggests an overall higher aggressiveness of males in this species and may explain why males, and not females, were targeted for the selection process. Differences were not only quantitative but the behavioural correlation networks differed between males and females of both strains, and for both the mirror and conspecific trials, confirming sexual differences in the pattern of aggressive displays. These results are in contrast with a previous study with commercial strains of *B. splendens* that described similar levels of aggression for males and females, both during mirror and live conspecific fights [43]. This divergence may be a consequence of the different strains used in the two studies. Although not very clear, it seems that the authors used long-tail *B. splendens,* selected for ornamental purposes, and in these strains it is possible that intersexual differences in aggression are low, maybe because overall aggression has been reduced during the selection process.

A third objective of our study was to contribute to the ongoing debate about the use of mirror tests to study aggression. The mirror test has been used extensively in studies of aggression, including in *B. splendens* (e.g. [23, 44]) but its choice is controversial for a number of reasons. First, some animals may be able to self-recognize in mirror images, a cognitive ability that was initially thought to be exclusive of humans but that has now been extended to many other mammalian and bird species [45] and even recently suggested for a fish species [46]. Second, the displays that are possible to be elicited with mirror images differ from those of live conspecifics. For example, antiparallel displays, where fish engage in head-to-tail lateral exhibitions, or “carrousels”, where fish chase it others tale simultaneously, are not possible [47, 48]. Third, the behavioural [49], endocrine [50] and transcriptomic [51] response has been shown to differ between mirror and live conspecific interactions.

The behavioral reaction of males and females of both strains to the mirror image or to the conspecific presentation were, in general, similar, paralleling previous results for fighting fish [52]. For males, the aggression score and activity-related behaviours did not differ between the two types of stimuli. Similar results were found for wild-type females while the exception were fighter females, which displayed more actively towards the conspecific than the mirror, suggesting that the live intruder was a more salient stimulus than the mirror image. Results for fighter females parallel a previous study in zebrafish that showed a more salient brain transcriptomic response to a resolved live interaction than to a mirror interaction [53]. The more intense response of fighter females to the conspecific interaction resulted in differences in the behavioural correlation network pattern between treatments, which were absent in the other groups. The reason why differences in the response to the two treatments were detected in fighter females but not in the other groups, may relate to variation in the behaviour of live conspecifics. Unlike the mirror trials, where the image matches the intensity and behavioural patterns of the focal animal, the conspecific presentation is more variable, with the model fish displaying different behaviours to those of the focal fish. This view was partially supported by our results that showed an overall higher variability in the response to the conspecific than to the mirror stimulus in fighter females but not in the other groups. Thus, it is possible that variation in the dynamics of the interaction with the live conspecific resulted in the observed differences in the response of fighter females to the two aggression-eliciting stimuli. Taken together, our results show that mirror presentations induced overall similar and less variable aggressive outputs to those of a live conspecific suggesting that the mirror test is as a useful tool for studies of aggression in *B. splendens*.

## Conclusions

The study highlights that long-term selection for male winners of staged fights ongoing in Southeast Asia with *B. splendens* has enhanced and modified male aggressive displays but also female displays. The results strongly suggest that in this species male and female aggression share common genetic and physiological mechanisms, opening testable hypothesis about its evolutionary consequences, further facilitated by the recent sequencing of the species genome [54] and transcriptome [55]. The study further contributes to the ongoing discussion about the use of mirror-elicited aggression tests in animal behaviour, showing overall similar responses to a mirror image and to a live conspecific.

## Additional file

Additional file 1: **Figure S1.** Frequency of frontal and lateral displays, caudal swings and charges for wild-type and fighter males (left) and females (right) during the conspecific and mirror trials. Numbers in columns indicate *N*. Means ± S.E. are shown. (DOCX 246 kb)

## Data Availability

All data is available upon request from the corresponding author.

## Abbreviations

*B. splendens*: *Betta splendens*
PCA: Principal component analysis
